# Multiligamentary Reconstruction in a Below-Knee Amputation Patient

**DOI:** 10.1155/2021/8854005

**Published:** 2021-04-06

**Authors:** María Tuca, Tomás Pineda, Ignacio Valderrama, Gonzalo Ferrer, Sergio Maass, Gonzalo Espinoza

**Affiliations:** ^1^Mutual de Seguridad, Santiago, Chile; ^2^Universidad del Desarrollo-Clínica Alemana de Santiago, Santiago, Chile; ^3^Hospital el Carmen, Santiago, Chile; ^4^Clínica las Condes, Santiago, Chile

## Abstract

Multiligament injuries in below-knee amputation patients are a severe condition, and its treatment is controversial. Its incidence is unknown, and it is highly underdiagnosed, representing a challenge for the physician. The case presented is about a patient with a left transtibial amputation secondary to a severe crushing of the ipsilateral lower limb to which during the process of physiotherapy, a multiligament injury was diagnosed. The patient underwent a tibiofibular fixation with a multiligament reconstruction with good functional results. In this complex situation, delay in diagnosis is frequent, ligament instability should always be suspected and explored further, allowing for proper rehabilitation and early treatment.

## 1. Introduction

The incidence of multiligament injuries in patients with transtibial amputation is unknown and be highly underdiagnosed, with delays in diagnosis for up to two years [1].

This can be explained by the fact that amputations usually occur in polytraumatized patients who have other multiple relevant injuries to take care of and with extended hospital stays which cause a delayed start in rehabilitation. Also, it becomes more difficult to detect instability through performing stability maneuvers since these patients have a shorter lever arm. Because of these reasons, identification of knee instability in below-knee amputee patients is usually delayed, and a high level of suspicion is recommended to avoid underdiagnosis [1].

Biomechanical studies have shown that there is an increased stress over the collateral and cruciate ligaments in patients with transtibial amputation who are gait prosthesis users, in comparison to healthy patients [2].

This would explain why the gait progression of these prosthesis users is usually difficult during rehabilitation, even in advanced stages of the recovery process [1].

## 2. Case Presentation

A 26-year-old male patient was involved in a motorized vehicle rollover and suffered a left transtibial amputation secondary to vascular injury and severe crushing of the left limb. As his rehabilitation progressed, six months after the accident, he reported knee instability when attempting gait with prosthesis.

Physical examination revealed a knee with mild effusion, range of motion 0-110°, and an undamaged stump. The stability tests showed anterior instability (Lachman Test (+) and anterior drawer test (+)), grade 3 varus stress test with no firm endpoint at 0° and 30°, and painful proximal tibiofibular joint instability. The posterior and medial stability were preserved. Functional scores were as follows: Lysholm 35 points, Tegner 3 points, and IKDC 39 points.

**Figure 1:**
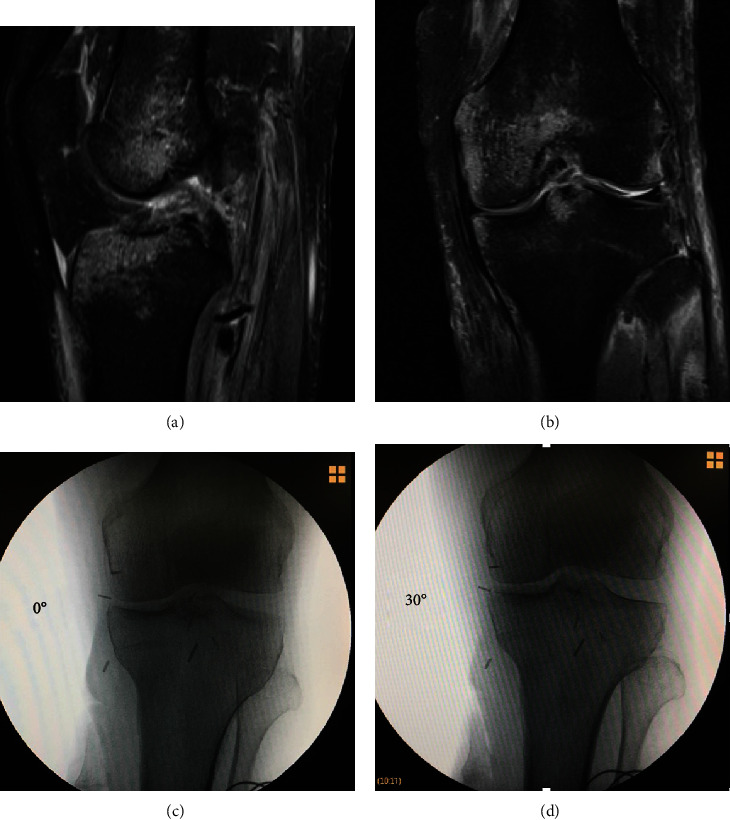
Preoperative images.

Magnetic resonance imaging of the knee showed a complete tear of the anterior cruciate ligament (ACL) (Figure 1(a)) and a high-grade posterolateral corner injury (PLC) (Figure 1(b)).

**Figure 2:**
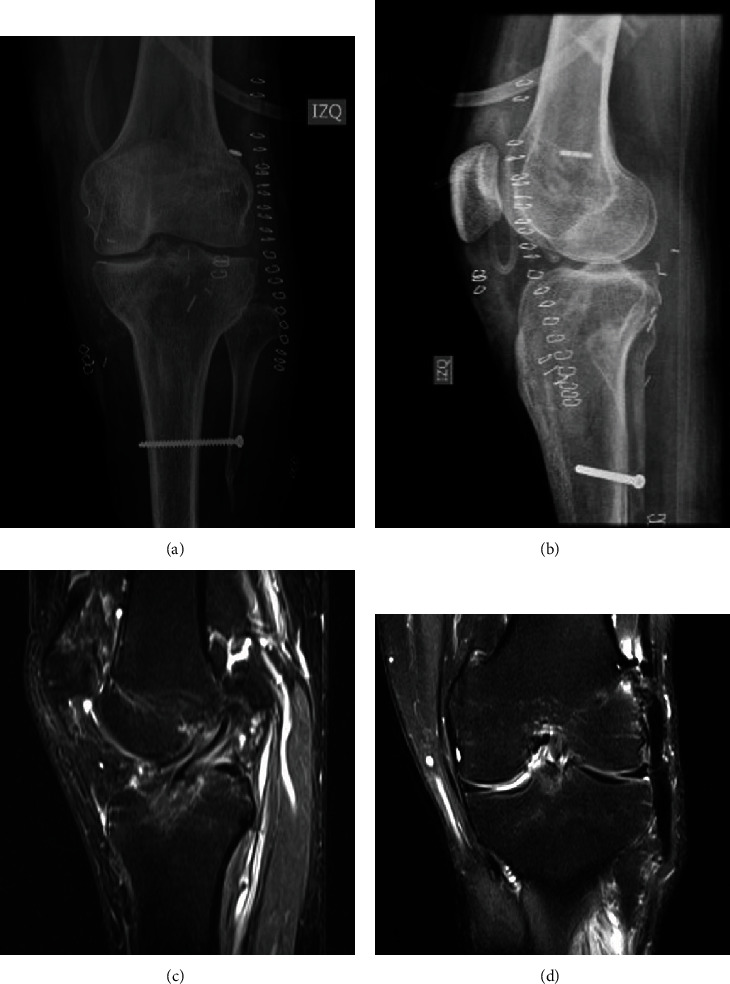
Postoperative images.

Diagnosis confirmation of the anterior, lateral, and tibiofibular instability was performed under anesthesia and fluoroscopy (Figures 1(c) and 1(d)). In a single stage surgery, the patient underwent a tibiofibular fixation with a screw (Figures 2(a) and 2(b)), an arthroscopic reconstruction of the ACL using peroneus allograft fixed with a cortical button in the femur and a bioabsorbable interference screw in the tibia (Figure 2(c)), and a reconstruction of the PLC with the modified Larson technique, using peroneus allograft and fixation with bioabsorbable interference screws in the femur (Figure 2(d)).

## 3. Results

Postoperative course was uneventful. At six months follow-up, gait progression was achieved with adequate prosthetic fit, without assistance and without subjective instability. Physical examination revealed anterior and lateral stability (IKDC A), with firm endpoint and full range of motion (0-140°). Functional scores at eight months after surgery showed improvements (Lysholm 81 pts, Tegner 3 pts, IKDC 63 pts).

## 4. Discussion

The management of ligament instability in patients with transtibial amputation is similar to that of patients with multiligament injuries and undamaged limbs, with some important exceptions.

Firstly, knee instability is tolerated worse in amputees due to the force ligaments that are subjected to in the context of gait with prosthesis, making stabilization surgery mandatory [1].

Despite growing evidence suggesting that stabilization surgery has better outcomes if performed early, in the context of amputees, as these are high-energy mechanism with large soft tissue damage, concomitant injuries, and a delayed diagnosis, in many cases, reconstruction must be deferred [3, 4].

In amputee patients, the graft selection and fixation method depend mainly on the surgeon's technique of choice. However, given the biomechanical alteration and the greater stress that the ligaments must resist in these patients, associated with a usually osteopenic bone after an extended unloading period, it is advised to prefer the use of cortical suspensory fixation systems (buttons) [2, 4].

As in our case, good results have been reported with the use of allograft in multiligamentary surgeries, which provide several benefits over autograft: multiple graft size options, less tourniquet time, no donor site morbidity, minor spectrum of associated injuries, and less probability of infection as there is no need of harvesting graft [1, 5].

Tibiofibular joint injury represents 1% of injuries around the knee and rarely occurs in isolation. It usually occurs in the context of bony and/or ligament injuries [6].

Its assessment and proper diagnosis are keys in the context of a PLC reconstruction, since reconstruction techniques use a tunnel at the fibular head level. Therefore, a stable tibiofibular joint is a must for a PLC reconstruction. An unnoticed tibiofibular injury can condition the success of the surgery [7].

The scarce literature on this association suggests that these lesions may be underdiagnosed.

In a retrospective study with 129 knees with multiligament injury, Jabara et al. reported a 9% incidence of proximal tibiofibular injury. They performed to these patients a ligament reconstruction together with a screw fixation, showing good functional outcomes in 32 months average follow-up [8].

In the case presented, proximal anteroposterior tibiofibular instability was diagnosed intraoperatively, which deemed PLC reconstruction as a necessary procedure. Tibiofibular joint was fixed more distal than usual with a 3.5 mm screw to leave space for PLC reconstruction, which achieved fibular stability in two points favoring the use of prostheses. However, to date, there is no evidence as to what height this fixation should be performed in amputees with multiligament injuries.

In conclusion, ligament instability must be suspected and thoroughly examined in patients with transtibial amputation, in order to avoid diagnostic delay and allow timely rehabilitation. Finding a possible associated tibiofibular instability is key to perform a successful PLC reconstruction.

## Data Availability

Case report: data of the patients are in the clinical records of the hospital.
